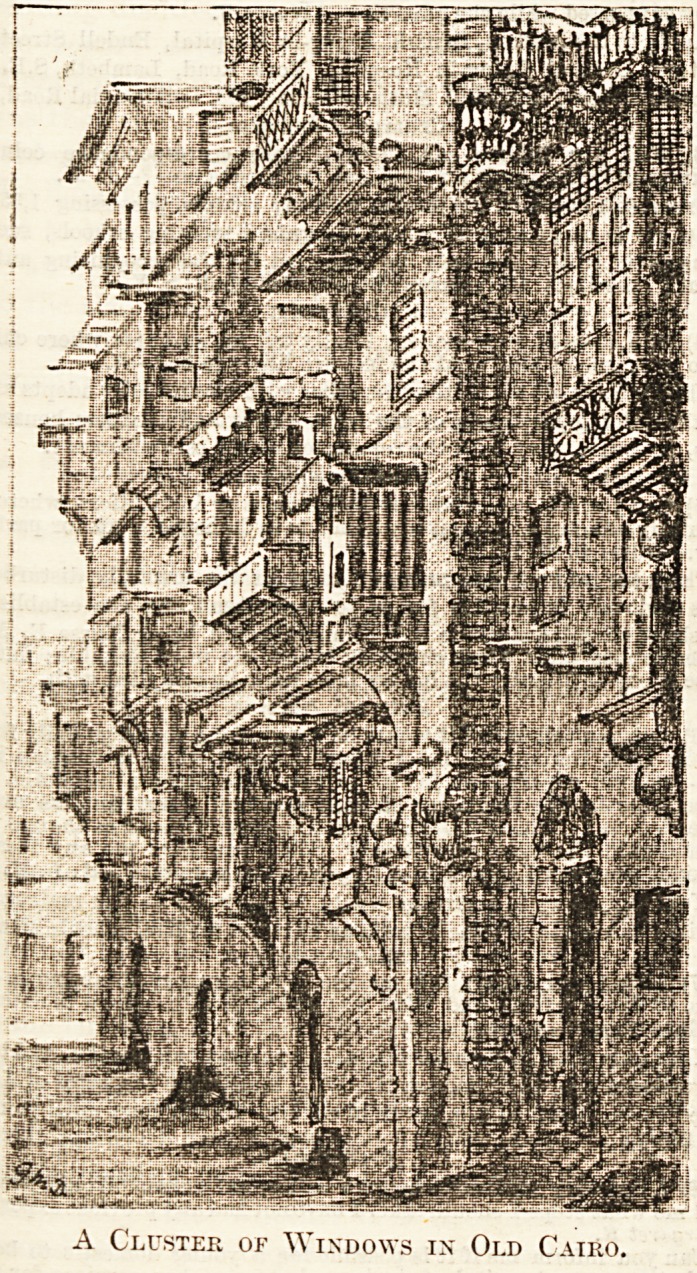# "The Hospital" Nursing Mirror

**Published:** 1899-04-01

**Authors:** 


### "The Hospital" Nursing Mirror.

Being the Nursing Section of "The Hospital."

[Contributions for this Section of "The Hospital" should be addressed to the Editor, The Hospital, 28 & 29, Southampton Street, Strand, London, W.C., and should have the word "Nursing" plainly written in left-hand top corner of the envelope.]

### To Our Readers.

It will be observed that the "Nursing Mirror" is considerably enlarged this week. The enlargement is permanent, for the demands upon our columns have increased so steadily that there was no other course open to us. With the fresh available space at our disposal, we shall be able to make room for New Features of Interest and Importance, which have been in contemplation for some time. One of these is introduced to-day in the shape of "Echoes from the Outside World," a page primarily intended for busy hospital nurses, but also meant for others who, from the nature of their duties, have little time to read the daily papers, or learn what is going on in the busy world. This is especially necessary since the circulation of the paper has so largely increased in the colonies and abroad among nurses, who, perhaps, do not see any other English journal, and who, as the letters we receive from time to time show, are only too glad to receive information regarding current events and the gossip of the day.

In early numbers we propose, among other items, to commence a series of Short Interviews, a series of Character Sketches of "Typical Patients," and A Matron's Corner, as well as to make a feature of special correspondence from the Colonies, under the title of "Across the Seas."

### Notes on News from the Nursing World.

### THE NATIONAL PENSION FUND.

The numerous nurses who attended the twelfth general meeting of the Royal National Pension Fund on Friday warmly welcomed the intimation that the Princess of Wales has expressed her gracious wish to present certificates to those nurses who have entered the Fund since the reception at Marlborough House in 1895. They fully appreciate the interest which is taken in their work by Her Royal Highness. Do they as fully realise the co-operative nature of the Pension Fund? For this is really a crucial point, and we are afraid that in spite of the reiterated emphasis which has been laid upon it many of them have still only a vague notion that the Fund was established and exists for their benefit. Whereas, of course, the truth is that they themselves are the Fund. It is for them to do what they like with their own, and nobody but themselves can derive any advantage from it. The number of proposals in 1898 was not unsatisfactory compared with the number of years which have elapsed since the Fund was founded, but it might, perhaps, have been larger if the policy-holders had sufficiently grasped and made known the cardinal principle that lies at the base of the society.

The reference in the report to sick pay touches upon the fact which we desire to emphasise. The Council last year, acting on the advice of Mr. King, the consulting actuary, strengthened the sickness account by transferring the sum of £2,300 from the "Council's reserve fund." It is inevitable that any undue advantage obtained in sick pay by an individual member can only be secured at the expense of the whole body. While the reduction of the "holiday" class, as distinct from genuine cases of ill-health, is a matter for congratulation, the Council must necessarily look to the policy-holders to encourage and assist them in their efforts to protect the interests of the many as against the doubtful claims of the few.

In the case of a society which has no directors' fees to pay and no shareholders to make provision for, it is obviously important that the working expenses should be kept down. On this matter the testimony of Mr. King, who is justly considered one of the leading authorities of the day on insurance, is very valuable. He declares that "they have reached the irreducible minimum." There are insurance agents who foster the idea among some nurses that the rates are heavy. In reality, even without bonus additions, the monthly rates of the Fund are lower than the lowest rates quoted by other offices; but with the expenses so low, it would not be of very much consequence if the premiums were high, because the whole of the surplus comes back to the policy-holder in the shape of bonus. Or, to put it in another way, the purchase value of one pound of premium is considerably greater than is shown by the tables.

### SYMPATHY AND SCIENCE.

The cause of trained district nurses needs no apology. We therefore pass over the excellent addresses and pleas for help put forth at the annual meeting of the East London Nursing Society by Prebendary J. F. Kitto (the chairman), the Rev. R. W. Harris, and others, and note only the statement of Sir William H. Quayle Jones and part of Prebendary Harry Jones' speech. This nursing association, according to the former, with a band of thirty nurses, ministers to a population of 350,000. It is impossible to conceive a stronger argument for liberal contributions to its support, especially when the surprise visit of the inspector of Queen Victoria's Jubilee Institute for Nurses resulted in her expressing herself delighted with the work. Prebendary Jones, happily defining trained nursing as "sympathy and science," called to remembrance Lieut.-Colonel Brackenbury's dismay during the Franco-Prussian War at the shoals of young ladies who offered their services as nurses to the wounded heroes. Their ideas on the subject of nursing were limited to the poetic view of sitting by their bedsides and smoothing their pillows, administering nips of brandy all round in between whiles! Sympathy was essential to nursing, but sympathy without science was often work thrown away. There was much that was tedious and disgusting in the care of the sick, and their trained nurses were practically acquainted with it all; nevertheless, continued Mr. Jones, knowledge had not blunted their sympathy, and their work was fully appreciated by their committee.

### THE PRINCESS CHRISTIAN'S TRAINED NURSES.

In glancing through the report of H.R.H. Princess Christian's Trained Nurses one is struck by the number of promotions that have fallen to the share of the nurses. The staff consists of a lady superintendent, two district nurses ("Queen's"), two probationers, and sixteen private nurses, as well as four district nurses residing in the parishes of Eton, Chertsey, Addlestone, and Egham, respectively. Of the seven private nurses leaving during the year, one has been appointed matron of the Windsor Royal Infirmary; another matron of the Ulster Hospital for Women and Children, Belfast; and a third sister in the Army Nursing Service. Amongst the district nurses one has been appointed Lady Superintendent of the Rathbone District Nurses' Home, Shaw Street, Liverpool. The financial year has been successful. Thanks to the exertions of friends, the first handsome instalment of £250 has been paid off the mortgage on the nurses' home. It has been found possible to reduce the expenditure by dispensing with the services of the assistant secretary. The Lady Superintendent, at the request of the Corporation of Windsor, arranged two courses of lectures to be given during the winter and spring. Mrs. Roberts, formerly one of the staff, undertook to deliver them, and handed over to the association the grant made by the town.

### SOUTHPORT'S SURPLUS GIRLS.

A correspondent, observing the report of Dr. H. H. Vernon, medical officer of health for Southport, that the female contingent of the popular Lancashire watering-place exceeds the male by more than 9,000, and that Dr. Vernon asks, in plaintive notes, "What shall we do with our girls?" suggests that some of them should be made nurses. We believe that the same suggestion was not only made in respect to Kensington a few years ago, where the spinsters were found to be out of all proportion to the married women, or the marrying men, but acted upon. Still, there is a limit to the demand for nurses, and our tip to the surplus girls of Southport is "Try domestic service." There is unquestionably an opening for lady helps with tact, who are not afraid of soiling their hands, and can cook a dinner daintily.

### THE STAFFORDSHIRE INSTITUTION FOR NURSES.

An interesting and gratifying feature of the twenty-seventh annual report of this excellent institution is that, in addition to the earnings of the nurses reaching the unprecedented sum of £5,194, the committee have been able to divide among them £566 in percentage. While the increase in the amount earned is partly due to the extra charges for some special cases, it is satisfactory to learn that it is chiefly the result of continuous employment. The staff has now reached the total of 97 private nurses, 15 district nurses, and 23 probationers, while no less than 789 cases, representing 3,627 weeks of nursing, received attention during the year. Miss Shirley, the lady superintendent, has the pleasure of feeling that her efforts to gain the confidence of patients and doctors in her own judgment, and in the skill of the nurses, have been not inadequately rewarded.

### SUDDEN DEATH.

The pitcher goes often to the well and yet is broken at last. Months pass, the routine of hospital life meanders on, lulling even those toiling in the midst of death into a feeling of false security, until at length one of the multitudinous accidents that are always possible happens, and, as in the case of Rochdale Workhouse, two useful lives are lost. Nurse Evans was apparently sitting at a desk in a small surgery at the Rochdale Workhouse taking an inventory of the bottles which Nurse Barker was counting, when the ether bottle slipped, and, falling against another bottle, the neck broke, the ether exploded, and set the place on fire. Nurse Evans expired almost immediately, whilst Nurse Barker lingered a few days in agony and passed away on March 21st. We tender our heartfelt sympathy to the relatives of these two ladies. Miss Evans, who was 41 years of age, was trained at St. Mary's Hospital, Manchester; Miss Barker was 35, and a native of Bradford.

### THE POSITION OF WOMEN.

Last week Mrs. Ayrton, wife of Professor Ayrton, delivered a learned lecture, the result of her own researches, before the Institution of Electrical Engineers to a large and enthusiastic audience, and in the last issue of the *Woman's Signal* the editress bids farewell to her readers, because she cannot spare the time to edit it from other important journalistic work. Public opinion now not only grants a woman full permission to exercise her talents to their utmost capacity, but there is a growing feeling—which some women do not care to observe—that it is her bounden duty to do so. Thus there are women doctors, lecturers, writers, artists, chemists, as well as nurses—the pioneer profession of the woman's movement. The contrast between to-day and 40 years ago is unwittingly emphasized in an article by Mrs. C. Tennant (in the publication just referred to) on Elizabeth Barrett Browning. She is in it reported to have expressed her opinion that the opening of nursing to ladies as a profession by no means solved the woman question, and that the evolution of women thinkers and artists would confer more benefit on general humanity. "The very same men," she remarked, "who called the lady nurses 'angels' would curse the impudence of the very same women if they stirred an inch as thinkers or artists." In the foregoing incidents and opinion the improvement in a woman's position between then and now is emphatically illustrated.

### TO NURSES

In order to increase and vary the interest in the *Mirror*, we invite contributions from any of our readers in the form of either a paragraph, a letter, or information, and will pay a minimum of 5s. for each contribution.

### SHORT ITEMS.

The last entertainment of the winter course for patients, 1898-99, took place on Thursday at the National Hospital for the Paralysed and Epileptic, Queen Square, Bloomsbury. Among the artistes were Miss Florence Oliver, Miss Violet Jourdain, Mr. Oswald Laxton, Mr. J. H. Curtis, and the wonderful child harpist, Winifred Hemming, seven years of age, who performed on both the Grecian and national harps.—The Secretary to the British South African Company, 15, St. Swithin's Lane, will be glad to receive contributions to the Dominican Sisters' Home in Bulawayo. The Administrator of Matabeleland, Captain Lawley, has been elected president. The home is to be erected as a token of the regard in which the service of the sisters to the sick in Rhodesia.

### Hints on the Home Nursing of Sick Children.

By J. D. E. Mortimer, M.B., F.R.C.S., formerly Surgical Registrar, &c., at the Hospital for Sick Children, Great Ormond Street.

(*Continued from page 263, Vol. XXV.*)

### HARE-LIP AND CLEFT PALATE—HIP DISEASE—CONVALESCENCE—TRAVELLING—SEA BATHING.

### Hare-Lip and Cleft Palate.

If a baby with either of these deformities is unable to suck, the mother's milk should be drawn off by a breast pump, and this or other milk given with a spoon, or from a bottle with a large teat, the hole of which has been cut out on the lower surface. Special teats are also made with flaps to close the opening in the palate. The baby must be fed while in the upright position, and the teat or spoon pushed well over the tongue. In case of hare-lip awaiting operation, the nurse should daily apply strapping by which the edges of the cleft may be brought nearer together and much improvement effected. At any rate, the gap is prevented from being stretched when the child cries or laughs. If the central bone projects its position may be considerably improved by gentle daily manipulation. After the operation the hands must not be left free to meddle with the mouth. The nurse should notice whether the baby is able to breathe without much trouble—it may be necessary for the surgeon to insert a tube into one of the nostrils. Any discharge should be wiped away, but the wound must not be wetted or otherwise interfered with.

### Cleft Palate.

It is a good plan for the nurse to have a few days with her patient before a cleft palate operation, so that they may get used to one another, and that she may impress upon the child, if he is old enough, that after it is done he must try and not speak nor cry, but make his wants known by signs. The temperature must be regularly taken, and the nurse should be careful to inform the surgeon if there has been vomiting, diarrhœa, cough, or any sign of ill-health such as may make it advisable to postpone the operation. Relatives (if in a private house) should be warned that it is essential for success that they should control their feelings, and on no account after the operation say or do anything which may excite the child or induce him to speak; in fact, it is better in many cases for them to keep away till the first week is over. When the operation has been completed the child must be put to bed, and will need constant watching and soothing. The head must be kept low at first, and lying on one side. On recovery from the anæsthetic there may be vomiting of blood which has trickled down. The child must not be allowed to put the fingers in the mouth (confine the hands if necessary). No solid food should be given for a week (particles of merely soft food are apt to cling about the mouth and decompose), only fluids by means of a feeder with rubber tube attached. The surgeon should be asked whether he wishes the mouth syringed. If possible the child should be taken out in the open air as soon as permission is given, but there must be no talking until the edges have firmly united. The nurse must not be tempted to interfere with or even to look at the wound except by the surgeon's directions.

### Hip Disease. Spinal Caries.

When treated by extension apparatus, besides the precautions necessary in all recumbent cases, the nurse must notice if the foot falls too much outwards, or points too much downwards; also if the lower part of the back arches up so that she can pass her hand underneath it, as in such cases some addition to the apparatus or some alteration in the pull may be needed. She must also take care that the heel does not get sore, and that the spreader is wide enough to keep the strapping from pressing on the ankle. For washing, &c., the child should be turned on the sound side, the nurse clasping the diseased limb just below the knee, keeping it steady and drawing it slightly away from the trunk in the direction of its own length, whilst the other hand is placed on the crest of the pelvis or haunch-bone (not the projecting trochanter of the femur or thigh bone, on which no pressure must be made at any time). The limb should then be supported by pillows, so that it does not drop on the bed—it must always be kept as if the femur were in one piece with the pelvis. If it is absolutely necessary to lift the child, the nurse should stand on the sound side and pass her hand nearest the child's feet underneath the sound leg, grasping the diseased limb as before; the weight having now been taken off, her other hand should pass under the hips, and she should stoop down for the child to clasp her round the neck, or an assistant may stand by her side and support the child by passing one hand under the shoulder-blades, the other under the head. If the child has to be put on another bed the change may be made whilst he is thus lifted, or the fresh one may be brought in a line with the old one (not to the side of it), and the bearers should take short side steps until he is in position to be lowered. Or a simple form of stretcher may be devised with moveable poles (and crosspieces to keep them at the proper distance apart), the canvas being put under the child as in adjusting a draw-sheet, and the limb steadied and supported as before. Similar precautions are needed in lifting or moving a child with spinal caries; the diseased part must be firmly supported and no strain put on it. In some cases of cervical caries (in the bones of the neck) the head has to be kept for months in a fixed position. A small pillow should be placed in the nape of the neck, and a large sand bag (not too full) should reach from one shoulder to the other in the form of a horseshoe round the head, being well moulded into the corners, and to this a handkerchief drawn firmly across the forehead should be fastened by safety-pins. On no account must the child be allowed to move the head, still less to sit up, until the surgeon gives permission. A sudden movement, especially a falling forward of the head, may mean instantaneous death from the upper part of the cord being crushed. Hence when changing a pillow, &c., the head and shoulders must be supported so that there is no movement between the one and the other.

### Convalescence.

It will very often—in fact, almost always—be found that the subsidence of acute symptoms is accompanied by an increase of irritability on the part of the little patient. There are many causes for this, some inevitable, but undoubtedly it often results from the child being allowed to get over-tired by too much talking, too long visits of friends, too many novelties, and so forth, and being allowed to attempt too soon games and occupations requiring effort or dexterity, when failure causes disappointment. The diet is apt to be too heating and stimulating, the bowels are usually sluggish, and with returning appetite there is likely to be over-indulgence, especially in regard to dainties between meals. Children should not be allowed to stand up, or even to sit up in bed, suddenly after having been kept lying down, as faintness may be caused. This is especially likely after exhausting diseases, but may occur even after a confinement of a day or two. The first walk or drive should be a short one, and a day chosen when there is no fog nor east wind. Clothing should be warm but not heavy, and care must be taken that the child is not kept about in a cold bedroom or draughty passage either when going out or on return. Infants are often chilled in this way and also by being allowed to play on the floor without being screened from the draught which comes under the door towards the fireplace.

TRAVELLING.

Details should be well thought out beforehand, and in case of a long journey there should, if possible, be a break. As breakfast is apt to be scanty on account of excitement a lunch or dinner basket is usually needed. Sandwiches (neatly cut and kept in cases or cardboard boxes) may be made, besides the usual meat, &c., with egg, thick preserve, cress or other salad, but no ham nor anything likely to cause thirst. Milk, lemonade, &c., should be put into screw-stoppered bottles, and pure water always taken, with some fresh fruit, a knife and saucer, also two or three small damp sponges and a towel. No stiff hats or bonnets should be worn, and some soft cushions, toys, and books are likely to be useful. Babies often travel most peacefully in a hammock slung between the luggage racks. It unfortunately often happens that the good done by a change to the country or seaside is to some extent nullified by want of proper sleeping accommodation, defective sanitary arrangements, and difficulty in procuring water and milk which is above suspicion. A furnished house is generally to be preferred to apartments, in which troubles with other lodgers and with the servants and landlady are so apt to arise when any special arrangements are needed for an invalid's benefit. No water should be drunk which has not been boiled or run through a Pasteur's or Berkefeld's filter. Milk should also be boiled or sterilised, or some good brand of condensed milk used. A child recovering from illness should be kept from over-excitement, even of a pleasurable nature, such as is apt to be induced at noisy seaside places. As a rule no active exercise should be taken in the afternoon, especially in summer. Care must also be taken to avoid over-exposure to the sun, such as is apt to occur when "paddling" at the seaside, the head being often quite inadequately protected by a flimsy hat or unlined sun-bonnet, whilst the blood is driven out of the feet and legs by the cool temperature of the water.

SEA BATHING.

The best time is two to three hours after a meal, and when the child is warm, but not overheated or fatigued. A few seconds in the water is quite enough at first. A short run afterwards, and a little hot milk or cocoa, will promote re-action, but if the extremities remain cold and bluish, or if there is headache or languor, the time of immersion should be shortened or the bathing stopped altogether, and sponging at home substituted, the child then standing in a pan of warm water. I need hardly say that young children should be allowed to accustom themselves gradually to bathing, and on no account be forcibly dragged in, and that after illness, especially when the heart or kidneys have been affected, the doctor should be asked if the child is fit for it.

### Life in a Plague-Stricken District.

AN interesting lecture on "Life in a Plague-Stricken District" was given by Dr. Marion Hunter, late plague medical officer at Poona, last week at 17, Pembridge Square, by kind permission of Dr. and Miss Gladstone, the proceeds being devoted to the funds of the Women's Industrial Council.

Dr. HUNTER could speak with the fulness of personal knowledge, for she worked at Poona during the thick of the last outbreak of plague, from November, 1897, to March, 1898. Briefly recounting the recent history of plague in India, from its first beginnings in 1896 through its decline, until there came a week in June, 1897, free from a single case, and the recurrence of the disease in epidemic form in the autumn of that year and the following spring, Miss Hunter went on to describe the means adopted by Government to check its spread. Poona was divided into wards, a census taken, and every means used to segregate and isolate, cleanse and ventilate. In December, 1897, a "health camp" was established, to which the natives were invited by every persuasive means, the idea being to empty the houses in advance of the plague, as it were, disinfect and whitewash, and so prevent its dissemination. Native unqualified practitioners were a great difficulty; they resented the attempts made to deal with them by the Government, and endeavoured by every means to stir up the people to resist the precautionary measures taken. By every device the natives tried to prevent the detection of plague cases; the dead bodies of their relatives they carried on to the roofs or buried beneath the floors of their houses. The General Plague Hospital was interesting as being the largest of its kind ever known, and the mortality within its walls was so comparatively low (56·6) that it was spoken of as the safest place in Poona. Here medical officers and nurses had their hands full indeed, admissions averaging at one time sixty per day, the staff consisting of three doctors and from five to seven English nurses. The difficulties of administration were largely increased by the native wards-men and women, who would sometimes all disappear in a body, or would steal the medicines from the different wards, or mix the various medicines together and drink them, while the impossibility of keeping the sick isolated from their friends added to their troubles. Dr. Hunter mentioned the fact that several cases of recurring attacks of plague came under her notice, and one patient was believed to have had no less than three attacks. The highest mortality was amongst the men, and the lowest amongst children, heart failure frequently causing sudden collapse, so that it was no uncommon thing to see dead bodies lying along the roads. Dr. Hunter concluded her address by giving an instance of the success of inoculation, and drawing attention to the undoubted fact that while Europeans seemed at first to be immune, the later outbreaks had proved increasingly fatal to them.

A very instructive discussion followed. Dr. Herbert Birdwood, C.S.I., gave some account of his own experiences of the plague in India; and Sir William Robinson, who was Governor of Hong Kong from 1891 to 1898, during which period he saw three plague epidemics, contributed many interesting details. Dr. Birdwood upheld the theory that plague was always worst in the dry season, and he urged that every effort should be made in the rainy seasons to stamp it out before the disease could reach epidemic proportions. The two remedies he specially advocated were (1) the complete draining of Bombay, as an enormous centre of infection and a city thoroughly water-logged; (2) the better ventilation of the streets and houses. Sir William Robinson said that in Hong Kong the worst outbreaks came in the hottest and wettest season, but it was true that they had followed on a time of great drought. In reply to a question as to the efficacy of treatment by vapour baths, Dr. Hunter said the treatment had not come into vogue at Poona to any extent, but she believed where tried it had induced very good results, diminishing delirium, lowering the temperature, and restoring consciousness.

### Wants and Workers.

MISS R. WEBSTER, Compton, near Guildford, Surrey, would be glad if anyone would give her a Surgical Aid Letter for a poor young man who has accidentally lost his left arm. She would be grateful for old linen or rag, as she is working in a poor country district.

### The Royal National Pension Fund for Nurses.

### REPORT OF THE TWELFTH ANNUAL GENERAL MEETING.

The twelfth annual general meeting of the Royal National Pension Fund for Nurses was held at the offices, 28, Finsbury Pavement, London, E.C., on Friday, March 24th, Sir Henry Burdett, K.C.B., presiding, in the unavoidable absence of the chairman of the council, Mr. Everard A. Hambro. Among those present were Mr. Edward Rawlings, Mr. Thos. Bryant, F.R.C.S., Mr. Gerard Norman, Mr. Chas. W. Trotter, Dr. E. C. Perry, Mr. W. S. M. Burns, Dr. G. W. Potter, Mr. Geo. King, F.I.A., F.F.A., Mr. C. Eric Hambro, the Hon. Egremont J. Mills, Mr. J. Pierpont Morgan, jun., Miss Monk, matron King's College Hospital, Miss Gordon, matron St. Thomas's Hospital, Miss H. A. C. Gordon, matron Charing Cross Hospital, Miss Haig-Brown, Miss Pritchard, honorary secretary Junius S. Morgan Benevolent Fund.

Mr. Louis H. M. Dick (secretary) having read the notice convening the meeting, the report and balance-sheet, which had been printed and circulated, were taken as read.

Sir Henry Burdett said: Ladies and gentlemen, I have now great pleasure in moving the adoption of the report. We have had a year of considerable progress, as shown by the figures. We find that 684 new policies have been issued for pensions producing premiums amounting altogether to £20,500. We have also had in the sick pay branch 161 extra policies, which represent annual premiums of £209 and a sick pay allowance in weekly payments of £123. Our investments have largely increased. They have gone up from £377,800 in 1897 to £441,344 in 1898. The investments of the Junius S. Morgan Benevolent Fund have increased from £16,700 to £17,100, and our total funds have increased from £394,500 to £458,414. There has also been a steady increase in the premium income of the Fund, which shows that the general prosperity of nurses is good; that income last year was £6,000 more than in 1897, and I need not remind you that when we started this Fund we hardly hoped to get so large a sum annually, whereas now we have a premium income not far short of £70,000 a year. I think it is a remarkable fact, which reflects the greatest credit upon our officers, that there has been a steady decrease in the percentage of the cost of management. If we had spent all the money which was provided for by the actuaries in their calculation we should have spent £1,600 more on working expenses than we actually have done during the last three years. But let me put it in another way. Our institution is a mutual insurance society, and when I tell you that the average expenses of management have steadily decreased every year from the first year, when they amounted to 8·2 per cent., to 3·6 per cent., at which they now stand, and that that figure is quite 1 per cent. less than the lowest rate of working expenses shown by any mutual insurance society in this country, I think you will agree with me that so far as economy is concerned we stand first amongst insurance offices.

### The Surrender of a Policy.

Another point which I want to bring out particularly is the question of surrenders. Every year a large number of nurses join the Fund and a certain number leave it. The actual money taken out by nurses leaving last year was £15,000. These surrenders, so far as the nurses are concerned, represent savings, with interest, which they would probably have never put by unless they had joined the Fund. So far as we are concerned, the surrenders are satisfactory from that and two other points of view. First of all, as a mutual society, it is essential that everything connected with our management should go to promote the stability and the prosperity of our members, and those nurses who surrender surrender necessarily before they have earned the maximum advantages, and consequently yield a profit to the Fund. The second is that all nurses who go out of the Fund before the pension age, as a matter of fact, add to the resources and to the profits of those members who remain in it. Analysing the surrenders, I find 20 per cent. were due to marriage, 30 per cent. were due to death or to nurses retiring from their profession because they had other resources or had made up their minds to leave it, 15 per cent. left because they were unable to continue their premiums, and 35 per cent. left for various reasons. When we come to analyse these various reasons chief amongst them stands the claims which are always being made by relatives upon thrifty members of the working classes, and under this head of "various" I think a large number (10) are due to the fact that the nurse determined to stand by her brother who was thriftless, and so to imperil her future, though she had taken steps to provide for it. I do think that this question of the claims made upon nurses by relatives should have the attention of the matrons of our hospitals and nursing institutions—some eminent representatives of whom I see before me—and of all who are interested in nurses, because it is a first principle that women especially who have to work for their living are not justified in depleting themselves to an extent which will leave them unprotected and unprovided for at a time when their working days are ended or when they may be disabled for work from other causes.

### Interest on Invested Funds.

Another point is the average rate of interest on investments earned by this Fund. Last year it was £4 2s. 3d. per cent., against £4 4s. 2d. for the last five years. We have also, owing to conversion and other causes, made a profit of £5,000. The invested funds of the J. S. Morgan Benevolent Fund, which amount to nearly £17,000, have earned no less than £4 6s. 9d. per cent. That high rate of interest is due largely—I may say mainly—to the fact that the relatives of the benevolent founder, Mr. Junius S. Morgan, are untiring in their endeavour to look after the welfare of the Fund, and it is by their efforts that we are enabled to earn so large a rate of interest. It is satisfactory in regard to the Benevolent Fund to say we have been able to give £120 more than ever before to aid members who were in temporary distress. The work of the Benevolent Fund has so increased that it has been found necessary to appoint a paid secretary. I think it only right to mention that when this fact became known the grandsons of the late Mr. J. S. Morgan came forward spontaneously and provided the secretary's salary, because they held that a Fund like this should have none of its resources devoted to management expenses. I am sure we are very much indebted to those gentlemen and greatly appreciate their liberality.

### Reception at Marlborough House.

It is now four years since the last reception at Marlborough House, and quite recently the Princess of Wales spoke to me about it, and expressed a wish that she should receive those members of the Fund who have joined since that occasion. There are upwards of 2,000 nurses entitled to receive their certificates at the hands of our President, the Princess of Wales, and it affords me great pleasure to say that Her Royal Highness wishes to receive them, and will do so so soon as arrangements can be made—whether it will be in the summer or in the autumn I cannot say at present. The secretary, however, desires me to state in this connection that nurses will be duly informed of the arrangements, and that it is not necessary for them to write to the office, as he is at present unable to give them any definite information. You will remember in connection with these receptions that the Princess of Wales was very anxious that there should be an armlet for the members of the Fund. That armlet is a very handsome badge, and it is very popular among nurses, but for reasons which are creditable to our policy-holders, and which point to the difficulties which they have to encounter, the armlets have not been generally worn. Nurses, like depositors in the Savings Bank, find it necessary for their own protection to keep their savings secret, otherwise they are the victims of attack, and there can be no doubt that the nurse who is known to have savings is beset by all sorts of people and receives all sorts of attentions which are given to her from motives other than those which may be regarded as strictly personal to herself, or which are due to the attractions which she may possess as an individual.

### Combine to Protect Your Interests.

I am very anxious to impress upon nurses that this Fund is a co-operative society. It belongs entirely to the nurses. It is established upon a commercial basis, and is conducted absolutely as a matter of business like any other insurance office, whilst its mutual character, the fact that the council and directors are unpaid, and that all the profits earned go direct to the nurses, places it beyond competition as far as other insurance offices are concerned. In connection with this mutual character I want to bring out that every member can help the Council and help herself by taking care, as the members of friendly societies do, that those members of the Fund who are entitled to sick pay do not unduly come upon the sick branch, and that it is confined strictly to those who are entitled to receive it through being incapacitated by real illness. That is a point I should like to bring closely home to nurses because we have found unfortunately that the demands upon the sick pay fund have been unduly large in the opinion of our actuary, and we are afraid that many nurses have come to think that when they are a little below par and want to take a holiday or go to a convalescent home it is very convenient to fall back upon the sick fund for the money for their holiday or change of air. That is what the Fund does not contemplate, and I hope nurses will bear it in mind, because it is impossible on any actuarial basis whatever to provide a fund which shall, in return for a reasonable premium, defray the cost of all the holidays which every member of the Fund may consider necessary or desirable whenever they are below par or feel they would like to take a rest. It is of the utmost importance that members should see that there is no malingering amongst nurses who are members of the Fund, and we hope to have the co-operation of our members in trying to support those—and we have many instances of it—who will not come upon the sick fund if they can help it. We ask our policyholders, therefore, to do all they can to persuade their sisters to confine sick fund payments to those who are worthy recipients owing to the nature and severity of the malady from which they are suffering.

### Great Growth in Profits and Bonus Distribution.

During the year we have had a valuation report from our actuary, which shows to a rather remarkable degree the progress which has been made by this Fund. During the first five years the surplus only amounted to £727 ; during the second five years the quinquennial valuation shows that the surplus has increased to £10,300. Then there is a further source of surplus, the Donation Bonus Fund, which has increased the total profits available for distribution with the object of increasing pensions, and which makes the total bonuses available £16,952. I am sorry to say that some nurses have such wonderful ideas about the power of our actuary, Mr. King, to show enormous surpluses that they have felt it would be possible in five years to make one pound into two pounds, and so forth. That, unfortunately, is not possible. But those members who have business friends will earn, if they consult them, that a result which in so short a time gives nearly £17,000 in profits, in addition to the interest allowed on investments, is remarkable, and it proves that this Fund offers advantages to nurses which they cannot obtain elsewhere.

### Improvement in Business Habits.

Perhaps it may interest you to know something of the work of the office. The number of receipts sent out last year was !9,000 ; the number of letters received amounted to 29,000, and the actual letters despatched from the office were not less than 31,000. I am happy to say in this connection that one marked result of this Fund is the improved business habits of the whole nursing body. It is very remarkable to look through the letters which we now receive, and to compare them with those which used to come in the earlier days. Now they are short, businesslike, and to the point, whereas formerly they did not partake of that nature, but occupied time in perusal and still more time in endeavouring to find out what the nurse wanted to know and the object with which the letter was written. I do think the matrons will be grateful to this Fund from the point of view that, apart altogether from its immediate object as a saving agency, it has done a great deal of good—thanks to the patience and tutorial powers of our secretary who has inculcated these habits of business—and has made our nurses not only thrifty but women of business too.

### Pensions Already Earned.

There is another branch of the Fund which is very interesting. We have existed long enough to pay pensions. Our object is to provide nurses with pensions, and I am glad to say that at the present time, though we have only been in existence some ten years, 255 nurses—those who are the older nurses and had the intelligence and courage to join this Fund early—are already in receipt of pensions amounting to £3,000 a year, which enable them to live, I hope, in reasonable comfort. If the present rate of increase continues, by the year 1900 we shall then be paying at least £6,000 a year in pensions to our nurses. It is interesting to note that the increase in the amount paid in pensions in 1898 as compared with 1897 is not less than 33 per cent.

### The Federation of Hospitals.

I want to bring out a point or two about federation. By federation, you know, I mean the plan which has been organised whereby a hospital or a nursing institution can recognise its duties to the whole nursing body by co-operating with this Fund and evolving a scheme whereby the whole of the nurses on their staff can be provided with sick pay and a pension. Very many of the best institutions have federated, and it seems to us that where federation is not yet taken up there is no one member of the committee who is sufficiently interested in the welfare of the nursing body to devote some little time and attention to elaborating a scheme which shall be acceptable to the nurses and which shall be just to them and to the institution. The secretary of a large hospital, who is often called upon to provide money for all sorts of objects, naturally does not care to work hard for federation. But wherever there has been a member of the committee who has had a wide intelligence and some feeling of responsibility, who has possessed a keen sense of what is due from the governing body to those it employs, there federation schemes have been successfully instituted without difficulty. I do hope there may be many gentlemen in this country who are sufficiently interested in the management of particular hospitals and nursing institutions to devote a little time to inquire whether their nurses are well protected by a scheme of federation. If they find they are not I am confident they will then exert themselves to secure that the omission shall be remedied without delay.

### The Case of Nurses in Ireland.

It has been said that Irish nurses are so underpaid or are in receipt of so small an income relatively to that received by nurses in other parts of the world that it is impossible for them to save. In the case of Dr. Steevens's Hospital, Dublin—thanks to the matron, Miss Kelly, who is a policy-holder herself—this Fund has been brought before the nurses, and eighteen have taken out policies in the Fund. I mention that to show where there is a will there is a way. For every nurse who begins to save in this Fund is in a much stronger position than if she keeps outside, and if not able to provide now the premium for the pension she desires at the age she hopes to retire from work, she should remember that by beginning to save what is possible she is putting herself in the best position for the future by entering when relatively young, and so getting a good investment. I believe if every Irish matron were to follow Miss Kelly's example the supposed difficulties in the way of Irish nurses joining would soon disappear, and we should have a very large number of policyholders from that part of the United Kingdom.

### A Word of Warning.

I do not think I should be doing my duty as your chairman if I passed over a feature which has been very apparent to us during the year. The agents of a small insurance company which has issued a special prospectus have been very active in trying to induce nurses to take policies in their office when desirous of becoming members of this Fund. Any nurse who yields to such blandishments will be likely to regret it at some time or other. Our Actuary prepared a report in which he showed, having regard to the nature and constitution of this Fund, its mutual character, the voluntary service of its council, the low rates of its working expenses and the largeness of its Bonus Fund, that it is impossible, as a matter of business, for any insurance office conducted wholly on commercial lines to give anything like the same return to nurses on their savings that this Fund does. The great insurance offices have recognised this fact, and have declined to compete with the Fund for nurses' savings. That fact should be known to nurses for their own protection, and I desire, with a full sense of responsibility, to caution nurses against taking out policies under conditions which cannot on any business basis result in anything but loss to themselves as compared with the return they must get if they join this Fund. Our Fund is essentially mutual and co-operative. It provides for the easy payment of premiums and allows nurses to pay them monthly, quarterly, or at irregular intervals as best suits their convenience. To those who are unable to meet the payments as they become due every consideration is shown, and time is allowed. The Fund further is an investment agency and a savings bank, for nurses can have their premiums returned to them with compound interest less a small reduction for working expenses. They can also pay in their money to a deposit account on which interest is allowed; every five years they have additions to their pensions arising from profits on the working of the Fund and also from the Donation Bonus Fund. They can insure, take out life policies, take out sickness and accident policies, and have as a friend and protector the Junius S. Morgan Benevolent Fund, which has always been able to help every nurse in the time of her distress, to tide her over that distress until she be restored, in God's good providence, to health, with the strength to resume her work.

I have endeavoured briefly to bring out some of the features of our work, and as far as possible to help nurses to understand the nature of the Fund, and to learn why, in our judgment, they would be wise to join. We are making steady progress, and the fact that our investments amount to nearly half a-million sterling within ten years is a surprising result, and should be a gratification to everybody, whether concerned in the management or as members of the Fund. In the Royal National Pension Fund we have not only the greatest thrift organisation for women workers in the empire, but an organisation which is as strong and as sound, in a business sense, as any commercial enterprise which the world has ever seen.

Mr. George King, F.I.A., F.F.A., had very great pleasure in seconding the adoption of the report, and said: After the comprehensive and lucid speech of the chairman it is not necessary that I should say much, and, in fact, it would be sufficient were I to merely formally second the motion. But there are one or two points it may be useful for me to touch upon. In the first place, I should like to mention the surrenders. The Chairman, in his address, spoke from the point of view of the Fund, and showed how those nurses who withdraw leave a profit to those who remain. But the matter may be viewed from the other side. There are many reasons for retiring, and where these are right and proper every consideration should be given to those who do retire; and it is so in this Fund. They receive their money back with an increase, and there is no risk of loss. The Fund thus becomes practically a Savings Bank. That seems to me to be a reason why nurses should join the Fund. Instead of holding back to see what her future means may be, a nurse can join and save her money, and later on, if she withdraws, take out a larger amount. Meanwhile it is perfectly safe. From that point of view, therefore, I think the system should draw the nurses to join, no matter whether they think they may not have the means or necessity of continuing membership. The expenses of management are remarkably moderate, and I think great credit is due to the council for its careful supervision and to the staff for the skilful way it carries on this organisation at so very little cost. That is really a source of profit from which the nurses who are members all derive benefit. The only point of real difficulty is the sick pay. I was very pleased that the chairman referred to that question in so emphatic a manner. I do not think the nurses intentionally do anything they feel to be wrong, but in the past there has been a want of knowledge as to the real meaning of this Sick Fund. They have not understood that it is meant simply for real illness that prevents work; and some have looked upon it as a kind of benefit fund to enable them when a little out of sorts to have a holiday. We heard of a nurse who wanted to learn to ride the bicycle, and said she would draw her sick pay a little longer for that purpose. It was not intended to be used in that way. This Sick Fund is intended for those who really require it, and no premiums would be sufficient to provide holidays of this kind. The Fund will work out satisfactorily if used for the purpose for which it is intended, but unless the nurses can be brought to understand its real nature it will not work properly, and the Fund will have to be closed. That would be a serious disaster, and I am sure it will not come about. But it is well to explain this point. The Sick Fund is for those who are ill—so ill that they are unable to work—and for no others. If these claim upon it the Fund is doing good work, and will be solvent; if others claim, the Fund will become insolvent. So far as I know, the Fund is the only one, on a large scale, which gives the benefits of friendly societies to women. The great majority of friendly societies are open only to the male sex. Here we have a fund open to women, and one which they should take full advantage of. The question of the valuation has also been dealt with, and it is fully mentioned in the report. It shows the prosperity of the Fund, because the valuations have been made on most stringent lines to preserve the Fund as a thoroughly sound, solvent institution. It is an institution with practically no cost of management beyond some out of pocket expenses, and the nurses who join will get all the benefits of their payments. That is not so with any other insurance company, and therefore nurses will do better to come here than go anywhere else. I have much pleasure in seconding the adoption of the report.

The motion having been carried unanimously,

Mr. GERARD NORMAN said : It is with very great pleasure that I propose the re-election of the following retiring members of the council : Sir Henry Burdett, K.C.B., Mr. Arthur H. A. Morton, M.P., Dr. Herbert P. Hawkins, and the Hon. Walter Rothschild, M.P. I am very sure that no recommendation on my part is necessary to ensure the unanimous re-election of these gentlemen.

Mr. WALTER S. M. BURNS seconded, and the meeting unanimously endorsed the motion.

Sir HENRY BURDETT proposed the re-election of Mr. Frederick Whinney, C.A., as auditor, remarking that the actuary's report showed he did his work very well.

This was seconded by Mr. RAWLINGS and adopted *nem. con.*

Upon the motion of the CHAIRMAN, seconded by Mr. CHARLES W. TROTTER, the Auditor's remuneration was fixed at 35 guineas, as before.

Letters of apology for non-attendance from several members of the council, and from matrons of various hospitals having been read, the report of the scrutineers was read, showing the following result : Miss F. C. Nott Bower, 1,147 votes; Miss Mabel Cave, 1,150; Miss Mary L. E. Dunn, 1,146; Miss E. Fisher, 1,145; Miss L. M. Gordon, 1,163; Miss K. H. Monk, 1,153; Miss E. Vincent, 1,138. Five ladies received two to eleven votes and ten ladies one vote each. The CHAIRMAN declared the seven ladies mentioned duly elected, and the voting cards were ordered to be destroyed.

Mr. THOMAS BRYANT, F.R.C.S., rose and said : It is my privilege to propose a vote of thanks to our chairman. I have much pleasure in doing so, for we are all much indebted to him. It must have been a pleasure to him to have seen the rapid growth of the prosperity of this association. I have been associated with it from the beginning, and if we had been told we could have reached such a state of prosperity I should have scouted the idea. Indeed, I never believed that an association for nurses based upon such principles could have been such a success. It is most gratifying to find that everything has gone on so well and we stand so well before the public. I propose a cordial vote of thanks to Sir Henry Burdett.

Mr. CHARLES W. TROTTER seconded, and the resolution was cordially adopted by acclamation.

Sir HENRY BURDETT returned thanks as follows : I have to thank you very much. I feel it includes the staff and my colleagues on the council, because there is a good deal of work in connection with this Fund. We very much appreciate the services of the secretary and those immediately under him. I should also like to say that the council very much appreciate the work of our actuary, Mr. King, and they congratulate him very heartily upon the soundness and accuracy of the way in which the Fund has worked out in practice and has attained a substantial financial position. I thank you very much indeed for the vote of thanks.

The meeting then concluded.

### Appointments.

UNIVERSITY COLLEGE HOSPITAL.—On March 25th Miss H. E. G. Hamilton was appointed matron of the above. She was trained at the Nightingale Home, and at St. Thomas's Hospital, where she afterwards became ward-sister and assistant matron. She was subsequently matron of the Cumberland Infirmary, Carlisle; and matron of the Victoria Hospital, Chelsea.

### The Association of Asylum Workers.

SIR JAMES CRICHTON-BROWNE, the president of the Association of Asylum Workers, took the chair at the annual meeting, which was held on the 27th ultimo at the rooms of the Medical Association, 11, Chandos Street, W. The report was taken as read ; the Chairman proposed its adoption, Miss Honnor Morten seconded, Dr. Needham supported, and it was carried unanimously. The association, the Chairman said, was neither moribund nor defunct; it showed, on the contrary, two unmistakable signs of healthy life—growth and metabolism. The roll of membership in 1897 was 2,534 ; in 1898 it was 2,890. The receipts in 1897 were £60; in 1899 £254. The balance at the beginning of 1898 was £82, and at the beginning of 1899 £137. All this was very satisfactory, but it was not just satisfactory enough. There were 10,000 asylum workers in England and Wales, and until the roll of membership numbered between 5,000 and 6,000 the committee could not be contented. It was expected that there would be a large influx shortly, because it could not be denied that at first the association had been regarded with some suspicion as a kind of trades unionism—an insupportable position for a band of Government workers. But now that the legitimate aims of the association were recognised, now that it was known to be under medical guidance, those who had stood aloof would fall into rank ; now that the workers realised that it was a co-operation for improvement of the social status of asylum workers it would become more popular. The second sign of health was metabolism, or change in constitution. The association consisted of a growing stable core, with a fluctuating unstable fringe outside. There was always a certain percentage of persons being drawn away from the work by more lucrative employment and other interests. There was at present too great metabolism; nevertheless the tendency was for the stable corps of workers to increase and for the unstable fringe to decrease. The Chairman said that he hoped great things from the settlement of the vexed question of pensions. It was not impossible that measures of legislation now before the present session of Parliament might result in a satisfactory solution of the problem. The wages, he contended, of asylum workers ought either to be largely augmented to enable them to make due provision for age, or a system of deferred pay—pensions—inaugurated, because, owing to the arduous nature of their work, the period in which premiums could be set aside for this purpose was very short, comparatively speaking. The former method was incomparably the more expensive. He believed that the asylum workers were as conscientious, as self-sacrificing, and as public-spirited a body of public servants as any ; but, as a contented mind is a continual feast, they ought not to be harassed and distressed by anxiety on their own account when the day of their weakness should come upon them. The education of asylum workers still occupied the close attention of the committee. For himself he had always held that the asylum attendant was a nurse and something more, and that when possible she ought to possess a year's training in a general hospital before undertaking mental work, or failing that she should have a full year in the infirmary ward under the instruction of a qualified superintendent. The possession of the certificate of the Medico-Psychological Association was very important. To those who remained in the work it was a passport to promotion ; to those who retired the knowledge gained was invaluable to those who had earned it. Commending to their notice the fund entitled "Homes of Rest" and the "Asylum News," Sir James Crichton-Browne finished a most interesting address with a warm tribute of appreciation to Dr. Shuttleworth, the hon. secretary, to whose initiative and energy the association owes its existence.

### Echoes from the Outside World.

### AN OPEN LETTER TO A HOSPITAL NURSE.

So you "feel very lonely away from us all," you poor dear, and "out of everything!" Well, the loneliness will soon wear away. People who are busy have no time to be lonely. There is nothing like good hard work to drive away the tendency to think about oneself. You will find that you cannot nurse your feelings and your patients at the same time, and so naturally your feelings go to the wall. But the other is to a certain extent a standing complaint. Nurses necessarily get little leisure, and when the time for recreation comes Wisdom generally decrees that they go out for a blow and a fresh air tonic. Thus the daily paper is bound to be neglected, and those who have liked to know how the world wagged when they were at home feel the difference and grieve over it. Therefore, I am going to write you a couple of columns each week, dear, and try to tell you any little thing which I fancy you would take an interest in, so that although you no longer live in the outside world you may hear its echoes in the sick room.

---

First and foremost, we are all being kept more or less busy just now with our yearly friend and constant visitor, Influenza. Those of us who are sufficiently fortunate to escape its ravages ourselves have to do the work of the invalids as well as our own, either in the home, the office, or the parish. Occasionally we hear of whole families all laid up at the same time. Then we have to play the Lady Bountiful, sail out with baskets of beef tea and jelly, and brave the fear of infection, to lend a helping hand. Otherwise, the wise ones are beginning to keep clear of an influenza patient as if he had the measles or the mumps. Not that such caution is always of use, for I see that lately the three occupants of a rock lighthouse on the south-western coast have all had influenza badly. The lighthouse is three-quarters of a mile from the shore, no one had been to land for a week, nor had any visitors been calling at the rock, yet first one and then another of the men fell a victim, though fortunately there was always someone well enough to trim the lamp.

---

But, happily, free from influenza myself, Saturday found me at the boat-race. I had not been for years, but the rumour of a probable contest between the two crews instead of a solemn procession sounded exciting, and we made up our minds early in the week to form a party and see the fun, unless we were snowed in, which would have surprised nobody. But you know what a change came over the spirit of our weather, and the sight of so large a crowd and (contrary to expectation), a fine day, was quite exhilarating. All who were sensible wore their thickest winter clothes, though one poor foolish girl in a pale blue silk blouse will probably have plenty of time during her coming attack of pneumonia to ponder over Cambridge's popular victory. I think everyone was glad that the tide had at last turned. Even the devoted adherents of Oxford must have become tired of foregone conclusions, and now the glories of the University boat race may return again. Is it not strange how history repeats itself? Nearly forty years ago Oxford won nine times in succession, then Cambridge scored, coached by an Oxonian. Again the mystic number has been reached, and again it is an Oxford ex-President who sends the Light Blues to victory. As a schoolboy with an enormous dark blue tie close to me on Saturday said with a complacent grin, "Well, if Cambridge has won, they'd never have done it if an Oxford man had not taught them a thing or two!"

---

There is no longer any doubt that England is going to take a big part in the Antarctic Exploration Expedition. Up to Monday last it seemed probable that owing to lack of funds other nations would be far before us, but at the meeting of the Royal Geographical Society it was announced that one member, Mr. L. W. Longstaff, had given £25,000, which, with the money already in hand or promised, ensures the dispatch of a British expedition. The Germans are making vast preparations, and will spend nearly £48,000, and with the aid of private explorers a great deal of an interesting character should be discovered, for, according to some authorities, the interior of the Antarctic is believed to be very different to the Arctic regions. At the North Pole, as far as have at present been discovered, all is snow and ice and frozen sea. At the South Pole many assert will be found a continent as big as Australia, with no Esquimaux and no bears. The great novelty in this forthcoming expedition is the employment of steam, which will make tasks once very difficult comparatively easy, although the exploration of eight millions of square miles (which is the measurement of the Antarctic circle) it is no trifling matter. One cannot but rejoice that such a munificent gift has been forthcoming, but I cannot help wishing that somebody equally rich and equally generous would come forward and help some of our poor hospitals. Just think what a difference it would make if only half-a-dozen wealthy men were to follow Mr. Longstaff's example.

---

I have been learning a few particulars which may interest you about the new magazine Lady Randolph Churchill is bringing out. I expect you know that the price is to be a guinea a number, and it will be published every three months. It is to be bound in dark green leather, tooled with gold; the paper has been specially manufactured for the purpose, so that it may be light in weight, and six photogravures will appear in each number, which Lady Randolph says will alone be worth the money. If advertisements can be made very artistic they will be allowed, but not otherwise. The name is to be *The Anglo-Saxon*, but American contributions will form an important item. Here, I think, Lady Randolph, who is sole proprietor and editor, shows her wisdom. She is more likely to find subscribers on the other side of the Atlantic than on this, I fancy, where guineas grow increasingly scarce. Anyhow, even the prospect of seeing our names printed at the end of this gorgeous volume among the subscribers will not induce you and me to order it through our local bookseller, so we must be content with a description only.

---

I know what an enthusiastic photographer you are. Here are two or three items I expect you would like to hear of. A new satellite of Saturn has been discovered—the ninth in number, but the first whose existence has ever been ascertained by means of the camera. A lady in Paris has lately commenced an action against an operator with the X rays. She had disease of the bones, and went to have the exact nature of the lesions demonstrated. She was examined three times, and at the last examination the operator scorched her so severely, she says, that he caused her to faint and did her great injury. The strange part of the case is that the Court knew so little about the matter that they had to postpone their decision till they had made some enquiries as to how such examinations ought to be conducted. Item Number Three—The carrier pigeons from Atlantic steamers are to carry messages on photographic films for the future. Each passenger who wants to send a message writes it on an ordinary-looking postcard with ten lines. A framework, capable of containing fifty-four of these cards, is placed before a photographic-reducing apparatus with twelve lenses, so that there are twelve copies for twelve different pigeons. Each proof consists of a film barely an inch and a-half square, but, nevertheless, containing the whole of the fifty-four messages. When the pigeons arrive at the home station these copies are photographically enlarged on special postcards and sent to the addressee enclosed in an envelope. Clever little arrangement, is it not?

### The Nurse's Part in Sanitary Science.

THE inclement weather kept many away from Mrs. Clare Goslett's address on "Sanitary Science for the Nursing Profession," delivered under the auspices of the Royal British Nurses' Association at 11, Chandos Street, W., on Friday. Dr. Wethered took the chair at six o'clock, and briefly introducing Mrs. Goslett, remarked that a nurse nowadays must know the underlying principles of ventilation and sanitation in order to be fully efficient.

Mrs. Goslett said that it was a great privilege to speak on a subject in which she was so much interested, not only to those whom she was addressing, but through them to the larger number to whom her remarks would be carried. We were now, she continued, at the end of a most wonderful century, and it was given to us to look back and see many changes—changes in which we may perhaps have had a share in bringing about. She would only note two, but they possessed advantages that would outweigh all others. The first was the emancipation of woman, the second the totally different way in which the public thought, and spoke, of disease. In past days people used to regard disease as a thing that must be, and epidemics as the dispensation of Providence. They had now learned that the ills they attributed to God were the result of ignorance. Whenever she wished to create a sensation in lecturing she referred to the epidemic of small-pox which was bound to come sooner or later, and no matter from what class her audience was drawn, the reference was received with horror, terror, and disgust. She bade her hearers remember how, at the present time, in the case of any sudden outbreak of illness such as typhoid, the question was indignantly asked, Who is to blame? Instead of apathy and indifference of former days disease was regarded as something someone ought to have prevented. This feeling was a great gain. Disease—especially infectious disease—was preventable, and why has its prevention not come about? This fact constituted the great claim of sanitary knowledge upon nurses. Sanitary science only came in with the Victorian era, though great medical luminaries of the previous reign had sought a preventive for gaol fevers, palsies, and kindred ailments. In 1798 Jenner had discovered a means to restrain the foulest disease, excepting leprosy, that had ever devastated the human race, and small-pox only exists to-day by the will of the people, or rather let us say through the ignorance of the people. But until 1837 there was no registration of births, marriages, and deaths—no Bill of any value to public health. The condition of the people, their method of storing refuse was inconceivable horrible; 3,000 persons out of 9,000 had no water supply, and purchased it from the carriers in the street at so much a bucket; baths were luxuries for the rich; the food was bad; life was hard and circumstances terrible. In 1848 came the first great Public Health Act, and perhaps nothing shows how its effects have permeated everywhere than the fact that every commercial person—even a sweep—is nowadays a sanitary person. No district nurse can do her work properly unless she has a knowledge of this Act, even the fact that she knows what is legal and can set the law in motion is a check to the scamped work of the jerry builder. The results may be seen in the death rate, which, from 75·5 per thousand then it has fallen to 18·0 per thousand now. Roughly speaking, that means a saving of 240,000 lives a year; and, what is of more importance, a saving of strength, vigour, and happiness to all. Typhus, yellow fever, dysentery, and ague have disappeared, and even cholera is baffled. The growing knowledge of sanitary science, or the great science of preventive medicine, has special claims on those who are doing such noble work in the cause of curative medicine, and in it women are given a free hand. A nurse must give some attention to the principle of this science if she is really to be up to date in her own sphere.

### Legal.

### CAN TRAINED NURSES DISCHARGE THEMSELVES FROM A CASE AT ANY MOMENT THEY CHOOSE WITHOUT NOTICE AND RECOVER THEIR WAGES?

A CASE interesting to nurses and to those who engage their services was heard at the Bloomsbury County Court, before his Honour Judge Bacon, the other day, in which the Nurses' Co-operation, 8, New Cavendish Street, sued Mrs. Cole, 42, Grove End Road, for the service of Nurse Belcher in a maternity case for one week at £2 12s. 6d. per week, and £1 11s. 6d. for waiting from October 13th to 22nd, when she was summoned to the case. The latter item had been tendered and paid into Court, and the defendant declined to pay for the week's service on the ground that the nurse had been engaged for a fortnight at the rate of £2 12s. 6d. a week, and had left at eight o'clock in the evening of Sunday, October 30th, without due notice. It was admitted on both sides that the hiring had been for a fortnight to attend the defendant's daughter in her confinement. The nurse stated that she had been discharged by the defendant and told to leave the house immediately, about two o'clock in the afternoon. This was denied by the defendant. The evidence showed that the nurse had partaken of dinner and had not left at two o'clock, but had gone out for her own purposes in the afternoon, returning about six o'clock, saying nothing whatever about her intention of leaving. About an hour later the nurse came down to the dining-room, and in the presence of witnesses informed the defendant that she had thought over what she (the defendant) had said to her in the afternoon, and she was going to leave as soon as she had made the patient comfortable for the night. Mrs. Cole expressed her astonishment and remonstrated with the nurse, as did also a gentleman, the defendant's solicitor, who happened to be a guest for the day. He cautioned the nurse that if she left in this way of her own accord she would forfeit her right to wages altogether, as she had been engaged for a fortnight. The nurse declined to discuss the matter at all and refused to stop, although she was aware that there was no other person in the house capable of taking care of the young mother or of the baby, except the defendant herself, and that there was no possibility of obtaining another nurse late in the evening, as it then was, and Sunday. His Honour Judge Bacon decided that upon her own evidence it was clear that the nurse had not been discharged by the defendant, but had discharged herself and broken her contract. Judgment was entered for defendant. A question had arisen early during the hearing of the case as to the right of the Nurses' Co-operation, who were stated to be an association of some 400 nurses, to sue. The defendant's counsel at once consented to the nurse's name being added as plaintiff. The plaintiffs were represented by Mr. Irving, barrister-at-law (instructed by Messrs. McKenna and Co., solicitors), and the defendant by Mr. Broadbridge, barrister-at-law (instructed by Mr. Green, solicitor).

### Minor Appointments.

THE COTTAGE HOSPITAL, DENNY, N.B.—Miss Elizabeth Hall, who was trained at the Huddersfield Royal Infirmary and at the Whitworth Hospital, Matlock, has been appointed Nurse-Matron of the above. She has been engaged as nurse at the Hammerwich Hospital, Staffordshire; the Memorial Hospital, Mirfield; and St. Leonard's Hospital, Sudbury, Suffolk.

RUDGWICK COTTAGE SANATORIUM.—On February 13th Miss Annie Leach was appointed matron of the above. She was trained at the Heywood Cottage Hospital, Burslem, and has since held the post of Sister at the Sheffield Royal Hospital for eighteen months.

QUEEN'S HOSPITAL, BIRMINGHAM.—Miss Lillias Pumphrey, who was trained at the above institution, was, on March 7th, promoted to the post of Theatre Sister.

### The East-End Mothers' Home.

BEHIND the unpretentious exterior of No. 396, Commercial Road, the East End Mothers' Home has carried on a good work for a number of years, and it is pleasant to record its recent extension by the acquisition, on lease, of the next house, No. 394. The increased accommodation has provided space for five additional beds, raising the total number to 18, and enabling the Home better to meet the constant and increasing demand upon its resources.

The new premises were "on view" last Friday, when the annual meeting was held at the Home, and very comfortable the wards looked; the convalescent "mothers" gathered round the fire, doing good credit to the excellent care with which they are surrounded by Miss Blomfield and her staff. The report presented at the meeting was in every way a satisfactory one; especially is the management to be congratulated on the fact that, although the number of patients admitted has much increased, and some have been seriously ill, yet no mother has died in the Home during the past year. The additional space, besides giving the nurses a sitting-room and a better dining-room than hitherto, eases the nursing arrangements considerably, the beds being now on two landings only, instead of scattered over the house. Miss Blomfield reported a good year's work with nurses and pupils. Nineteen midwifery pupils were trained, of whom all but three gained the L.O.S. certificate, and 22 maternity nurses successfully completed their terms at the home, and earned its certificate.

During the past year 255 in-patients were admitted, and 293 out-patients were attended at their own homes. With regard to finance, the committee invite special donations this year towards reducing the bank loan, which stands at £1,100. The cost of the recent building improvements and repairs amounted to £313, to meet which the bank loan was somewhat increased, and though the interest payable is equivalent to a rent for No. 396, to purchase the freehold of which the money was originally borrowed, it is greatly desired that the amount shall be steadily reduced. It would be almost impossible to over-estimate the good which is done in this crowded East End district by the mere existence of such an institution as the Mothers' Home, not only from the point of view of the individual mother, who receives as much care and attention during her confinement as her richer sisters can command, but in consequence of the all-important lessons in cleanliness, and the management of their own and their babies' health, which the women learn during a stay in the home, and which are inculcated in poor homes by the midwives and nurses. The committee's request for continued and increased help will appeal especially to all who have any knowledge of the aims and achievements of the East End Mothers' Home. A personal visit will be the surest way to convert those who now know it only by name into sympathisers and supporters.

### Novelties for Nurses.

### GOOD SCOTCH WINSEYS.

THE good qualities of winsey are not as much realised nowadays as they should be. It is an excellent material when well manufactured, being light, soft, firm, and durable. Messrs. James Spence and Co., of Dundee, have sent us a charming set of patterns most suitable for spring and summer wear. We advise nurses to write and ask that some may be sent to them, that they may judge for themselves how suitable the materials are to their use. They will wash well, a valuable quality in itself. Some of the soft fine white winseys should certainly find a place in the layette. Others equally fine, and with silk stripes, are most suitable for blouses, whole costumes, or bed and tea jackets. Plain-coloured materials would serve for the nurse's uniform; the quality is excellent and the price most moderate. Readers should write to Messrs. Spence, of Dundee. The firm has no connection with that of the same name in St. Paul's Churchyard.

### The Bookworld for Women and Nurses.

[We invite Correspondence, Criticism, Enquiries, and Notes on Books likely to interest Women and Nurses. Address, Editor, THE HOSPITAL (Nurses' Book World), 28 & 29, Southampton Street, Strand, London, W.C.]

NOTES ON SURGERY FOR NURSES. By J. BELL, M.D.Edin. Fifth edition, revised. Pp. 194. (Oliver and Boyd. 1899. Price 2s. 6d.)

A BOOK by Dr. Joseph Bell—and in its fifth edition, too—stands in no need of the reviewer's praise. But we must say of this book that it is of the purest gold; it contains nothing that nurses should not know, it omits little that they need know. But Dr. Bell's book is really a book for nurses, and one which pays them the high compliment of recognising that they are not inferior practitioners, but members of a grand profession, separate in aim and independent in dignity. The concluding chapter of "Advice" is pungent—almost caustic. But the nurse who reads it intelligently cannot fail to gain increased pride in her own work, and to cease to think it fine to usurp the functions of others.

INFATUATION. By R. M. CROKER. (Messrs. Chatto and Windus. 1899.)

"INFATUATION" is a thoroughly nice story. It is well written in a vigorous style. The characters are well drawn, and there are not too many of them to entangle the thread of the tale. One could have wished that the gentle, intelligent, and lovable heroine had had a little more decision of character, but had this been the case there would have been no story. Maria Talbot loves young Borrodaile from the time she is in the school-room, and he, a selfish, dissipated fellow, amuses himself during his furloughs (enforced and otherwise) by exercising all the charm of this personal beauty and ingratiating manners in fostering this childish predilection. It becomes at last an infatuation which well-nigh ruins her life. Believing Maria an heiress Borrodaile proposes, and the wedding is fixed, but only to be postponed indefinitely. For by the sudden death of Maria's father it is found that all her fortune excepting £60 a year has been lost in speculation. Captain Borrodaile returns to India. His letters grow fewer by degrees until, under the influence of other flirtations, they finally cease. Poor Maria, now the unpaid companion of a selfish and tyrannical aunt, wastes the best years of her life in patient fidelity to a man who throws her letters unopened into the waste-paper basket and in cherishing a dog "Hero," his gift to her. For he never asks his freedom, although he meets and recognises her, himself unknown, until he hopes to win an American heiress, a charming girl, with wits, energy, beauty, and pluck for a dozen. Then, and then only, he sends her his card with the initials "P. P. C." pencilled on the corner. It is a terrible blow to the patient woman, but she is no fool, and once convinced of his unworthiness she puts all thought of him away. Towards the end there is a graphic scene, where he seeks her to plead her silence on the past, and finds her, estranged from her aunt because she will not leave her dog to die alone, beside that dog's body. The American, who has become Maria's fast friend, arrives in time to hear a part of the conversation, and with incisive scorn expresses her opinion of him and to him in no measured terms. Baffled and humiliated, he appeals to Maria for sympathy, but she has only one word for him, "Good-bye." Of John Harland and his faithful love, of their final happiness, of the success and fortune that come to both, the reader will learn as, with unflagging interest, he follows the story to the last page. It is just the book for both convalescent and nurse when they are tired and want amusing without any exertion on their own part. There are no obtuse problems, no harrowing "realistic" descriptions of social blots and blemishes, no wearisome introspection and discussions. It leaves a pleasant impression on the mind, and at the same time has kept it well entertained.

### Everybody's Opinion.

[Correspondence on all subjects is invited, but we cannot in any way be responsible for the opinions expressed by our correspondents. No communication can be entertained if the name and address of the correspondent is not given, as a guarantee of good faith but not necessarily for publication, or unless one side of the paper only is written on.]

### THE STAFF-NURSE AND HER PROBATIONERS.

"EXPERIENTIA" writes: I should like to call the attention of your readers to a subject which, I venture to think, is of considerable interest and no little importance. I refer to the duty of the staff-nurse towards her probationers. A staff-nurse is obliged to report to the matron the work performed by, and the progress of, her probationers, and it is with this particular portion of the staff-nurse's duty that I am at present concerned. I fear that reporting frequently degenerates into mere tale-bearing and an unnecessary repetition of those small shortcomings from which even the best of us are not free. The staff-nurse is often too eager to report the mistakes of a probationer when such a report can serve no good ends. This may, of course, be from a mistaken idea of her duty towards her superior and also of that towards her juniors, and I trust this may be so in the majority of cases; but, alas! too often, I fear, she is actuated by other and less interested motives, and the staff-nurse hopes by an exhibition of false zeal to curry favour with the matron. But I regret to say that this is by no means the worst. The staff-nurse will not only diligently carry to head-quarters every petty fault and trivial error, but will not infrequently bestow upon one or more of the probationers a large amount of favouritism, and having thus placed a probationer under a considerable obligation, will expect her in return—even if she does not instruct her to do so in so many words—to report to her the sayings and doings of her fellow-probationers when her back is turned. "We speak that we do know, and testify that we have seen." Favouritism is bad at any time, but combined with such a system of espionage its consequences are baneful in the extreme. Jealousy, mistrust, and suspicion take the place of love, friendship, and confidence amongst those whose work should be their bond of union, while the effect of this on the mind of a young girl may readily be imagined, and doubtless many a one has had her life made unhappy and her mind warped by living in such an atmosphere.

[We insert the above with reluctance, because whilst "Experientia" has touched a point the discussion of which may produce good, yet it nevertheless requires unbiased judgment to discriminate between espionage and oversight, fair report and tale-bearing. What would be espionage and tale-bearing in the fellow-probationer in the staff nurse is oversight and honest report.—ED. *T.H.*]

### Dorset County Nursing Homes.

### THE COST OF A NURSE.

A PARTICULARLY instructive correspondence on the cost of a trained nurse is appearing just now in the columns of the *Dorset Chronicle*. It was begun by a country parson who was somewhat indignant at the charges of the association and because one of the nurses objected to sleeping in the cottage of her patient. Lady Baker's letter on the subject is pertinent, and we, therefore, quote it:—

"May I, in connection with the County Home for Nurses, draw your attention to one or two facts connected with your late correspondent's letter? First, that the usual cost of an ordinary charwoman varies from seven to ten shillings a week and her food, while the cost of a nurse, trained for not less than a year, varies from 15s. a week to £2 for a fully-trained one. We think, therefore, that if we supply a nurse at less than half her average cost, and for little more than the cost of a charwoman, we are not doing badly for the poor. It is not supposed that they can afford to pay all this themselves; but for what are our weekly offertories for the sick if not to help in such cases? And surely only subscribers, as Captain Acland says, have any right to ask for a nurse at less than half her cost. This, by our rules, in cases of extreme poverty we may always grant. Secondly, to give an idea of the difficulty of obtaining nurses at all, we have all been ransacking London for trained nurses of any sort who will come for £35 or £40 a year (all found) into the country so far from town, and we cannot get them for love or money. If we send half-trained women to dangerous cases we are blamed, and rightly, by the medical men. If we send trained nurses (who will, of course, do no menial work) we are blamed by the parson and the poor themselves. The real truth is, the poor don't want as a rule to be nursed, and would rather risk their lives in the hands of their relations; but is that any reason for neglecting them? The wealthy, on the other hand, or many of them, are chary of unbuttoning their pockets unless they can be sure of a twopenny loaf for one-halfpenny. It comes to this—as long as our subscription list only makes up half the cost of the home we can only afford a corresponding reduction. Directly we have sufficient income to meet the absolutely necessary expenditure the poor can be nursed free. With regard to the Ockley system, as its originator is a personal friend of mine I will not expose its weak points; but I have gone carefully into the working of it at its source, and find it inadmissible in our already overcrowded cottages. With regard to the question of a bed, I have constantly found that the nurse is expected to occupy the only recently vacated bed (in the daytime), much after the fashion of Box and Cox, and this your correspondent will allow is scarcely to be expected even of trained nurses.

"AMY BAKER,

"Late hon. secretary of the Dorset Health Association."

### For Reading to the Sick.

### EASTER JOY.

RISE, heart! Thy Lord is risen! Sing His praise without delays,
Who takes thee by the hand, that thou likewise with Him mayst rise;
That, as His Death calcined thee to dust,
His Life may make thee gold, and much more just!

—*Herbert.*

Thou knowest He died not for Himself,
Nor for Himself arose;
Millions of souls were in His heart,
And thee for one He chose.
Upon the palms of His pierc'd hands
Engraven was thy name,
He for thy cleansing had prepared
His water and His flame.
Sure thou with Him art risen; and
Now with Him thou must go forth,
And He will lend thy sick soul health,
Thy strivings might and worth. —*Keble.*

So should we live that every hour
May die as dies the natural flower—
A self-reviving thing of power;
That every thought and every deed
May hold within itself the seed
Of future good and future meed;
Esteeming sorrow, whose employ
Is to develop, not destroy,
Far better than a barren joy. —*Houghton.*

The resurrection of Jesus Christ from the dead is the great pledge which He has given man of His immortality. St. Paul declares the Christian belief in the words, "Now hath Christ been raised from the dead, the first fruits of them that are asleep." The first fruits are fruits to be followed by further fruits of a similar kind—the pledge and earnest of the coming harvest. The belief that death does not end all, but is to be succeeded by a future life, is assured by the resurrection and ascension of Jesus Christ, and by His positive teaching. He distinctly taught that death is not the end of life, but only an accident in life; and that the river of life flows on in the same course which it took before it passed the cataract of death.—*Staley and Illingworth.*

The practical instincts of pure affection and noble aspiration point imperiously to a better world.

As certainly as sleep implies awakening so the grave means resurrection from the dead, means that here we work and there we wait, wait for the great awakening; this is the solemn mystery of the grave.—*Knox-Little.*

### Travel Notes.

By Our Travelling Correspondent.

### XVI.—EGYPT.

Fifty years ago a journey to Cairo was looked upon as a serious undertaking; the daring traveller made his will, set his house in order, and took solemn farewell of his friends; but now all is changed, and a six months' sojourn in Egypt is embarked on as lightly as a trip to Paris; everything is easy, luxurious, and agreeable, the only drawback being the amount of money required.

### Various Routes to Egypt.

The land route viâ Dover, Calais, Paris, Milan, and Brindisi is the most expensive, but is the best for bad sailors or for those who like to travel in a leisurely manner with frequent

A Cluster of Windows in Old Cairo.

halts. It costs, 1st class, £23 4s. Then there is the P. and O. steamer which goes viâ Gibraltar and Malta in twelve or thirteen days, fare, 1st class, £20 16s. 6d. This is a good route if you are a good sailor, for there is no hurry and excitement over changes, and the long sea voyage is in many cases beneficial. If you are bound for Alexandria you will change steamers at Brindisi. A third way, and the cheapest of all, is from Liverpool to Ismailia by Anchor Line; fare, £13 14s. 6d. There are several other ways of reaching Egypt, and I shall be happy to give any further information desired.

### Money and Passports.

The exchange of money is not profitable, as in some Continental countries English sovereigns and piastres (five of the latter to a shilling) are the coins in constant use, and small copper coins called para for baksheesh. It is well to be bountifully supplied with these latter. Have some French money with you also, as it is used sometimes in Alexandria and in Cairo in some of the pensions and shops. Take your money in circular notes, there is no other arrangement so convenient, and it is absolutely safe if you keep the notes and the "letter of indication" in separate receptacles. Passports are a necessity in Egypt and can be obtained easily, price 3s. 6d., through Messrs. Gaze or Cook before leaving England.

### Expenses for Those in Easy Circumstances.

As my good housekeeper would say, "there's no going away from it" that travelling in Egypt needs a long purse. You will manage very cleverly and economically if you live in Cairo for three months, see the ordinary sights, and cover unavoidable expenses for £100, and that will not include steamboat or dahabeyah excursions, of which more anon. If you go for pleasure as well as health, and naturally wish to see everything as far as possible, your expenses will far outstrip that sum, to say nothing of the £50 or £60 consumed by the journey to and from England.

### For Those of Straitened Means.

In the case of health alone being the cause of the journey, and means being limited, a residence in Cairo might be managed for three months on £50 by staying in a pension, and if the cheap journey by Liverpool is adopted, costing there and back with extras £30, the whole expenses might be covered by £100 without pinching. This is only possible by life in a pension. I have no personal knowledge of them in Cairo, but am told that they are comfortable and well managed, though your common sense will point out that for a sum of from eight to twelve francs per day the luxuries to be met with at Shepheard's will be lacking. There are several reasons against apartments. Primarily, it is difficult to get them for a short term, and housekeeping on your own account, unless you know a little of the native language, would be very difficult, probably resulting in much waste of money and temper. There are hotels as low as 10s. per day for a prolonged stay, but extras will run the bill up, and on the whole an unpretending pension at from eight to ten francs is the best place for those to whom economy is important. If it is necessary to live outside Cairo—and, of course, it is desirable for invalids—the lowest terms at the Mecca House Hotel, eight miles south of Cairo, are from 12s. to 14s. per day. Always bear in mind that it is poor economy to take an invalid from all his home comforts and plant him in the midst of mean surroundings. Take a few days whilst sumptuously residing at Shepheard's to study the pros and cons before permanent settling anywhere.

### Clothing Required.

The air is bland and delightful, but the sun very hot, necessitating precautions against its ardour; the mornings and evenings are, however, sometimes decidedly chilly; you must, therefore, be well supplied with woollen underclothing. Because you feel very hot do not be tempted to throw aside wool and take to cotton; it is seriously dangerous. The clothing may be light, but that it should be of wool is a necessity. Even to a robust person a chill, probably contracted after great heat, is not to be disregarded, and sometimes induces an obstinate kind of feverish attack by no means easy to cope with. Clothing is expensive in Cairo, so have your things new. For three months you will need two tailor gowns of different thickness; a coat and skirt is the best style, because it admits of wearing flannel blouses for ease and comfort and the addition of silk waistcoats for smartness. The frocks should be light in colour, for the dust and sand soon makes dark materials look shabby. Tan shoes and stockings are advisable. They are certainly cooler, moreover you can clean them easily yourself. Have two wraps, such as a substantial golf cape and alpaca or silk dust coat. Sailor hats with rather large brims are good; also the frightful but useful puggaree should not be forgotten.

MEDICINES AND USEFUL ARTICLES.

Quinine will often check a feverish attack, and whilst taking it try to induce perspiration by hot baths and drinking the juice of a lemon in a tumbler of hot water. Sunstroke and diarrhœa are two unpleasant dangers to be apprehended, though a little care will generally ward them off or at least neutralize their effects. For the former always protect the back of your head and neck. Never mind looking ugly; that is preferable to being ill. If diarrhœa threatens take first a little castor oil, and then arrowroot with small doses of opium in it. If it continues call in a doctor; it is not safe to trifle with this symptom if continued.

Arnica will be useful to anoint the bruises you secure in exploring the antiquities, and ammonia for the stings of insects.

Do not forget an india-rubber hot water bottle nor the invaluable tea basket.

### TRAVEL NOTES AND QUERIES.

PARIS PENSION (A Traveller).—Thank you for the addresses sent. I am always glad to have any with a good personal recommendation. I cannot give the address in THE HOSPITAL, as that would constitute an advertisement, and must be paid for as such, but I will gladly note addresses both in Paris and Naples, and add them to my list.

CENTRAL ITALY (Kodak).—No, do not take apparatus for developing, as you will be so constantly on the move; the spotting was probably caused by damp. The heavy white mists which you encounter in the mountains are fatal to films. The same thing has happened to me. Have a waterproof case for your Kodak, and keep it in a drawer or cupboard. The Italians are not so "difficile" about photographers as Germans, still, you must exercise caution near the frontier.

NAPLES (Enquirer).—It is not advisable to try second class hotels in Naples, and as you will only be there a few days best go to Parker's. The excursion to Vesuvius is very exhausting and disagreeable, and hardly repays one in interest.

SWITZERLAND FOR SKETCHING (Nemo).—It is difficult to advise you where all is so fine. You have to choose from amongst the chief centres—Zermatt, Martigny, Grindelwald, Interlaken, or Meisingen—or taking Lucerne as a starting point. The grandest scenery does not always lend itself to the best sketch. The Brunig Pass is lovely, and not too vast for effect. The Brunig Hotel is a delightful resting place, but Meisingen would be cheaper, and you would be nearer to the lovely flowers on the road to Rosenlain and the great Schiedegg.

LOWER BRITTANY (Cyrano).—Most delightful, but if your companion is at all delicate I should not recommend it, because, though the hotels are substantially good if you are strong, there is nothing suitable for invalids either in accommodation or food. Write me again further.

BIARRITZ (Evening Star).—Fare, first class, viâ Dover and Calais, £6 8s. 9d. The season lasts from July to October ostensibly, but in reality it begins much earlier. If you do not mind wind, Biarritz is delightful in the spring. It is rather exposed, and therefore not well suited to those suffering from lung trouble, but for anæmic patients and those requiring an exhilarating climate it is admirable. The hotels are all good and all expensive, and living is decidedly dear.

ARCACHON (Fiddler).—You ask which is preferable, Arcachon or Bournemouth, and I say most unhesitatingly Arcachon. In the lust for building the woods have been cut down round and in Bournemouth which were so beneficial to the consumptive. Arcachon stands in the midst of pine forests still. Fare, first class, £5 13s. 6d. Living is not very expensive for a long stay.

ITALY IN THE SPRING (Stella).—Nothing can be more delightful. Start first through the Italian lakes, then Venice, Florence, and Siena and Perugia. You would be a little too late for Rome, I think. Better to leave it for another year. Spring is *the* season for Italy. Much trouble will be saved by making out your tour and getting tickets from either Cook, Gaze, or Dr. Lunn. It is not a gain in money, but a very great save in trouble.

(*For Travel Advertisements see Page* xix.)

### Pension Fund Nurses.

MISS BURNS' WEDDING GIFT.

WE have to acknowledge the following contributions and to announce that our list is now finally closed, and that no more contributions can be received: Policy 5,544, M. M. Williams, G. B. Macvitie, Policy 5,520, A. Alloway, Policy 3,181, A. Petty, Policy 1,238.

### Notes and Queries.

The contents of the Editor's Letter-box have now reached such unwieldy proportions that it has become necessary to establish a hard and fast rule regarding Answers to Correspondents. In future, all questions requiring replies will continue to be answered in this column without any fee. If an answer is required by letter, a fee of half-a-crown must be enclosed with the note containing the enquiry. We are always pleased to help our numerous correspondents to the fullest extent, and we can trust them to sympathise in the overwhelming amount of writing which makes the new rules a necessity.

Every communication must be accompanied by the writer's name and address, otherwise it will receive no attention.

*Medical Congress.*

(1) Kindly tell me, through the medium of your paper, where and when this year the Medical Congress is to be held.—*Riviera.*

The International Medical Congress will be held next year in Paris. It usually meets every third year.

*Maternity Training.*

(2) Can you kindly tell me how to obtain maternity training for a capable young widow (27), who has a delicate baby (three months) to maintain, and no means? A small premium might be raised, or her baby supported while she gave time.—*Nurse B.*

Apply to the Matron, British Lying-in Hospital, Endell Street, W.C. Matron, General Lying-in Hospital, York Road, Lambeth, S.E.; Lady Superintendent, East-End Mothers' Home, 396, Commercial Road, E.

*Crumpsall Infirmary.*

(3) Will you inform me through your correspondence columns if Crumpsall Infirmary is a recognised training school?—*L. M.*

Crumpsall is one of the largest training schools, possessing 1,350 beds. As there is at present no list of recognised training schools, such questions as the above can only be answered by the recognising authority, namely, the Local Government Board, Whitehall, S.W.

*Chiropody.*

(4) Will some of your readers kindly tell an ex-nurse where chiropody is to be learned; also what the fees are likely to be?—*R.*

Chiropody is often practised together with manicure by adepts attached to the toilet departments of the large stores and business houses. The fees, of course, vary with the reputation of the establishment.

*Mentally Afflicted.*

(5) Can you tell me of any refined homes or institutions where ladies mentally afflicted are received and made to do house work for part of the day.—*Nurse E. M. C.*

There are innumerable private homes for the mentally disturbed, but it is obviously impossible for us to recommend any one establishment. A short advertisement in our columns would bring Nurse E. M. C. a dozen replies, from which she could choose the most suitable; but if the case is one of insanity the lunacy laws must be complied with.

*External Nursing.*

(6) Can you tell me if it would be possible for me to go daily for a few weeks from the nurses' hotel where I board when in London into the out-patients' department in one of the large London hospitals, to gain experience in the treatment of such accident cases, &c., as are there attended to? Which hospital should you recommend as the one where I should gain the most experience? Could you give me any idea of the amount per week I should have to pay to the hospital?—*H. L. M.*

Your only way to ascertain would be to write to the matrons of the various hospitals, especially such as have not medical schools attached.

*South Africa.*

(7) Would you kindly tell me the best means of obtaining a case to take to South Africa, and what steps to take with regard to entering an asylum there?—*Nurse.*

Your only plan is, in order to obtain a patient, to advertise, and to watch advertisements. Write and ask Mr. W. J. Dodds, the Inspector of Asylums, Cape Town, South Africa, for information. Do not start until you receive his reply.

*Probationers.*

(8) I am very anxious to become a hospital nurse. Would you kindly tell me what steps I should take. I have not had any previous experience.—*Margaret R.*

Can you inform me if it is possible for a young domestic to become a probationer at a hospital, if she is ready to give her services for a stated time without paying probationer's fees? And if so, what would be the probable cost of her uniform, clothing, &c.?—*Auriol L.*

Both our correspondents should obtain copies of the "Nursing Profession: How and Where to Train," price 2s. (The Scientific Press, London, W., publish it).

*Midwifery.*

(9) If there is any book or books that would be useful for me to study preparatory to entering Queen Charlotte's Hospital? I have already a good knowledge of physiology and hygiene. 2. Is there any demand by the various nursing associations for certificated monthly nurses without any general training?—*Annis.*

Haultain's "Handbook of Midwifery," 6s. (The Scientific Press, W.), is one of the best. 2. There is always a demand for monthly nurses, but it depends greatly on the connection and recommendations of each nurse. Most nursing institutions now require a hospital training as well as the L.O.S. certificate with any nurse admitted to their staff.

### ANSWERS REQUESTED.

*Nursing in Upper Egypt.*

(10) Now that Egypt is being opened up, will you kindly let me know if there is any one part of the country beyond Cairo more suited for invalids than another? What months of the year are best suited for travelling there? Are there many travellers in that country? and what nursing homes have they for invalids?—*Cairo.*

## Figures and Tables

**Figure f1:**